# Effect of Polymer Demixed Nanotopographies on Bacterial Adhesion and Biofilm Formation

**DOI:** 10.3390/polym11121921

**Published:** 2019-11-21

**Authors:** George Fleming, Jenny Aveyard, Joanne L. Fothergill, Fiona McBride, Rasmita Raval, Raechelle A. D’Sa

**Affiliations:** 1 School of Engineering, University of Liverpool, Liverpool L69 3GH, UK; sggflemi@liverpool.ac.uk (G.F.); zippy78@liverpool.ac.uk (J.A.); 2 Institute of Infection and Global Health, University of Liverpool, Liverpool L69 7B3, UK; jofoth@liverpool.ac.uk; 3 The Open Innovation Hub for Antimicrobial Surfaces, Surface Science Research Centre, University of Liverpool, Liverpool L69 3BX, UK; fmcbride@liverpool.ac.uk (F.M.); raval@liverpool.ac.uk (R.R.)

**Keywords:** antimicrobial, nanotopography, polymer demixing, biofilm, biointerfaces

## Abstract

As the current global threat of antimicrobial resistance (AMR) persists, developing alternatives to antibiotics that are less susceptible to resistance is becoming an urgent necessity. Recent advances in biomaterials have allowed for the development and fabrication of materials with discrete surface nanotopographies that can deter bacteria from adhering to their surface. Using binary polymer blends of polystyrene (PS), poly(methyl methacrylate) (PMMA) and polycaprolactone (PCL) and varying their relative concentrations, PS/PCL, PS/PMMA and PCL/PMMA polymer demixed thin films were developed with nanoisland, nanoribbon and nanopit topographies. In the PS/PCL system, PS segregates to the air-polymer interface, with the lower solubility PCL preferring the substrate-polymer interface. In the PS/PMMA and PCL/PMMA systems, PMMA prefers the air-polymer interface due to its greater solubility and lower surface energy. The anti-adhesion efficacy of the demixed films were tested against *Pseudomonas aeruginosa* (PA14). PS/PCL and PCL/PMMA demixed films showed a significant reduction in cell counts adhered on their surfaces compared to pure polymer control films, while no reduction was observed in the counts adhered on PS/PMMA demixed films. While the specific morphology did not affect the adhesion, a relationship between bacterial cell and topographical surface feature size was apparent. If the surface feature was smaller than the cell, then an anti-adhesion effect was observed; if the surface feature was larger than the cell, then the bacteria preferred to adhere.

## 1. Introduction

By 2050, it is predicted that the rise of resistant strains of bacteria and the ever-growing threat of antimicrobial resistance (AMR) will be the cause of 10 million deaths annually, and will burden the global economy by £64 trillion [1]. One of the leading factors for this surge of resistant superbugs is the misuse of current antibiotics in both clinical and agricultural settings [2,3,4,5,6]. Further problems arise when bacteria are able to irreversibly attach to a surface, which leads to microcolony formation and eventually biofilm maturation [7]. Indwelling medical devices, such as catheters, stents, dental implants, and orthopaedic prostheses, are susceptible to bacterial adhesion and frequently incur biofilm-associated infection [8,9,10,11,12,13,14]. Biofilm-associated bacteria are significantly more difficult to treat compared to their planktonic counterparts, with some estimates of their tolerance to antibiotics to be between 10^2^ and 10^3^ times higher [15,16,17]. As such, it is important that alternative solutions to combat biofilm-associated infections are developed.

A promising alternative to current antibiotics and disinfectants in combatting biofilm infections is by physically altering the nanotopography to form surfaces unfavourable to bacterial adhesion. This strategy of developing anti-adhesive surfaces takes inspiration from nature: shark skin is made up of tooth-like micro-scales that promote low drag and do not allow fouling organisms to attach to the surface [18]. The nanotopography of the lotus leaf consists of small cone-like protuberances that result in a superhydrophobic surface. On these surfaces water droplets remain spherical and pick up bacteria and other contaminants, as they roll off [19]. Surface topoography can even be bactericidal as in the case of the cicada wing in which sharp nanopillars pierce the bacterial membrane [20].

Surface topography-based strategies are gaining popularity as alternatives to or to work in synergy with chemical strategies to impart an antibacterial effect. One particular methodology that has economic advantages compared to more expensive techniques, such as electron beam lithography, is polymer demixing. This technique uses the immiscibility of two polymers in a common solvent. Upon spin coating, the polymer blend spontaneously undergoes vertical then lateral phase separation to give thin polymer films with discrete nanotopographies. Recently, this technique was used to investigate the topographical effects of nanoislands on cellular response [21,22,23,24,25,26].

In this paper, we fabricate polymer demixed films with discrete nanotopographies from a range of binary blend systems using polystyrene (PS), polycaprolactone (PCL) and poly(methyl methacrylate) (PMMA) polymers. PS can be molded into a large number of materials due to it being a viscous liquid above its glass transition temperature. It has medium to high tensile strength, however it also has poor chemical, oxygen, UV resistance, and low impact strength, which can all be overcome by copolymerization. PCL is a biodegradable polymer that displays excellent low temperature flexibility and toughness. PMMA displays excellent light transmission, is tough, durable and lightweight and also has high resistance to UV and weathering. PCL has found uses in clinical applications such as orthopaedic coatings, sutures and drug delivery systems, while PMMA is used as materials for dental implants, bone cements, and intraocular lenses [27,28,29,30,31]. By using the polymer demixing process in this study, we were able to assess the nanotopographical effect on bacteria, while the underlying surface chemistry remained constant. Bacterial adhesion of *Pseudomonas aeruginosa* (PA14) was used in this study to develop a fundamental understanding of how topographical approaches using clinically relevant polymeric materials can pave the way to designing antibacterial (anti-adhesive) surfaces that will resist biofilm formation.

## 2. Materials and Methods

### 2.1. Preparation of Polymer Demixed Thin Films

Glass coverslips (13 mm in diameter) were cleaned by immersing them in 5% NaOH for 30 min, followed by concentrated HNO_3_ for 30 min. Coverslips were then washed in EtOH (4 × 2 min), rinsed in DI water and dried at 80 °C. Coverslips were then treated with oxygen plasma for 1 min. PS (M_w_: 280,000), PCL (M_w_: 48,000–90,000) and PMMA (M_w_: 350,000), all purchased from Sigma Aldrich, were dissolved in chloroform (CHCl_3_) to give 5% w/v stock solutions. 1% w/v binary blend solutions of PS/PCL, PS/PMMA and PCL/PMMA were made by diluting and mixing the stock solutions in the following ratios: 0:100, 25:75, 50:50, 75:25 and 100:0. Aliquots of the blend solutions (70 µL) were spin coated onto freshly cleaned coverslips at 4000 rpm for 2 min, using an SCS P6808P spin coater (Specialty Coating Systems Inc., Indianapolis, IN, USA). Spin coated films were dried in a vacuum desiccator at room temperature overnight, to allow evaporation of any remaining CHCl_3_.

### 2.2. Contact Angle Analysis

Static contact angles of water were used to determine changes in surface wettability following each step of the synthesis, using an Attension ThetaLite optical tensiometer (Biolin Scientific, Västra Frölunda, Sweden). The sessile drop method was used, and contact angles were taken at 17 frames per second for 10 s and data recorded using OneAttension software (Biolin Scientific, Västra Frölunda, Sweden). At least three readings were performed per sample type and the results recorded as the mean average ± standard deviation.

### 2.3. Fourier-Transform Infrared Spectroscopy Analysis

Fourier-transform infrared (FTIR) spectra were obtained at room temperature in the spectral range between 3200 cm^−1^ and 1400 cm^−1^, using a PerkinElmer Frontier FTIR Spectrometer (Perkin Elmer, Llantrisant, UK). The spectra were obtained with 64 scans at resolution of 4 cm^−1^ and data was collected using PerkinElmer Spectrum v10.4 software (Perkin Elmer, Llantrisant, UK).

### 2.4. X-ray Photoelectron Spectroscopy Analysis

X-ray photoelectron spectroscopy (XPS) analysis was carried out on an Axis-Supra instrument (Kratos Analytical, Manchester, UK) using a monochromated Al Kα X-ray source operating at a power of 225 W. Charge compensation was performed using a low-energy electron flood source. Survey and narrow region scans were carried out at pass energies of 160 and 20 eV and step sizes of 1 and 0.1 eV, respectively. Hybrid lens mode was used in both cases. Data were converted to vamas (*.vms) format and analysed using CasaXPS 2.3 software (Casa Software, Devon, UK). Spectra were calibrated to 285.0 eV, corrected with linear background removal and fitted using Gaussian–Lorentzian line curves.

### 2.5. Atomic Force Microscopy Analysis

Atomic force microscopy (AFM) was used to observe changes in surface topography occurring during synthesis. A Bruker Multimode 8 (Bruker, Billerica, MA, USA) system fitted with a NanoScope V controller was used, and samples were imaged in air in ScanAsyst mode using a silicon RTESPA-150A tip operating at a scan rate of 0.9 Hz. Third order flattening was used to correct any errors while processing the image. 5 × 5 µm^2^ images were taken and root mean square roughness (*Rq*) and average roughness (*Ra*) were calculated from at least three replicates of each sample type, from at least three points per sample and measured using NanoScope Analysis 1.7 software (Bruker, Billerica, MA, USA). All data is presented in mean average ± standard deviation.

### 2.6. Adhered Cell Colony Forming Unit (CFU) Assay

Antimicrobial tests were carried out against the *P. aeruginosa* laboratory reference strain, PA14, a highly virulent strain of *Pseudomonas aeruginosa* (*P. aeruginosa*) with strong biofilm-forming capabilities [32] Overnight cultures of *P. aeruginosa* were diluted to McFarland Standard 0.5 in Luria-Bertani (LB) broth which is equal to approximately 1.5 × 10^8^ CFU/mL. Substrate disks were placed in 24-well plates and 2 mL of the bacterial solution added before incubating at 37 °C for 24 h to allow biofilm formation. At the end of this time, substrate disks were transferred to sterile well plates and washed three time with Phosphate Buffer Saline (PBS) to remove any non-adhered planktonic bacteria. Substrates were then placed in fresh wells with fresh media and agitated vigorously, by pipette to remove and re-suspend the attached biofilm. A serial dilution was performed on LB agar using the Miles and Misra method in order to enumerate the bacteria from the biofilm [33]. All samples were studied in triplicate and repeated five times. The results were recorded as the mean average ± standard error CFU/mL.

### 2.7. Statistical Analysis

The statistical analysis of bacterial numbers was performed using the data analysis package SigmaPlot 13.0 (Systat Software, San Jose, CA, USA). One-way analysis of variance (ANOVA) was used to establish differences between group means and thus variance between treatment types. Significance between treatment types was determined using the Student–Newman–Keuls (SNK) method. A value of *p* < 0.05 was taken as statistically significant.

## 3. Results

### 3.1. Wettability: Contact Angle

The wettability of the surfaces was determined using static contact angle analysis. The contact angles of polymer demixed films are recorded in Table 1. PS_100_ exhibited the greatest hydrophobicity, with a contact angle of 83.8°. A lower contact angle was observed for PCL_100_ and PMMA_100_ with values of 76.2° and 68.9°, respectively. For both PS/PCL and PS/PMMA demixed films an increase in contact angle was observed as the concentration of PS increased. As the concentration of PCL increased in the PCL/PMMA demixed films the contact angle too increased, showing an expected decrease in wettability.

### 3.2. Surface Chemistry: FTIR

**Figure 1:**
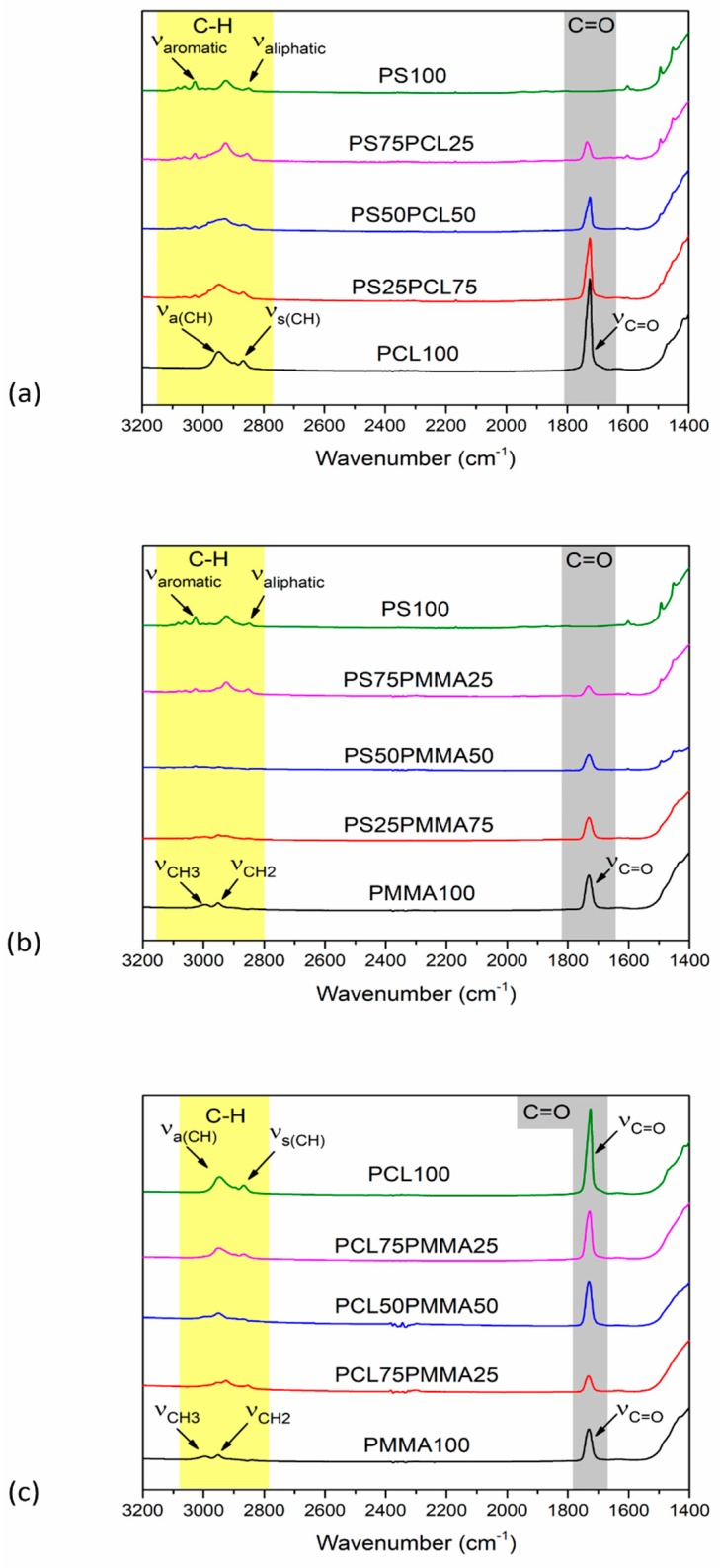
FTIR spectra of (**a**) PS/PCL (**b**) PS/PMMA and (**c**) PCL/PMMA demixed films.

The surface chemistry of the polymer demixed films was determined using FTIR and representative spectra are shown in Figure 1. The resulting spectrum of PS_100_ exhibited peaks at 3026 cm^−1^, due to the aromatic C–H stretching vibration (ν_aromatic_) and at 2925 cm^−1^ due to the aliphatic stretch (ν_aliphatic_). PCL_100_ was characterised with bands at 2948 cm^−1^ and 2865 cm^−1^ due to asymmetric (ν_a(CH)_) and symmetric CH_2_ stretching (ν_s(CH)_), respectively and at 1726 cm^−1^ due to carbonyl stretching (ν_C=O_) [25]. Bands observed in the PMMA_100_ spectrum at 2996 cm^−1^, 2950 cm^−1^ and 1726 cm^−1^ were characteristic of CH_3_ stretching (ν_CH3_), CH_2_ stretching (ν_CH2_) and carbonyl stretching (ν_C=O_), respectively [25]. In PS/PCL films (Figure 1a), as the concentration of PS increased, the intensity of the carbonyl peak decreased and there was a subtle shift in the peak from 1726 cm^−1^ to 1730 cm^−1^. This relatively small shift in the carbonyl absorption band has also previously been observed by Mohamed et al. with blends of PCL and PS [34]. In PS/PMMA films (Figure 1b), as the relative concentration of PS increased, the emergence of a band at 3026 cm^−1^ was attributed the aromatic C–H stretching of PS. In all spectra, except that of PS_100_, the carbonyl stretch specific to PMMA was observed. In PCL/PMMA films (Figure 1c) when the concentration of PCL increased the band was downshifted from 1732 cm^−1^ (PCL_25_PMMA_75_) to 1728 cm^−1^ (PCL_75_PMMA_25_), as now the carbonyl band contribution is higher from PMMA in comparison with PCL [25].

### 3.3. Surface Chemistry: XPS

**Figure 2:**
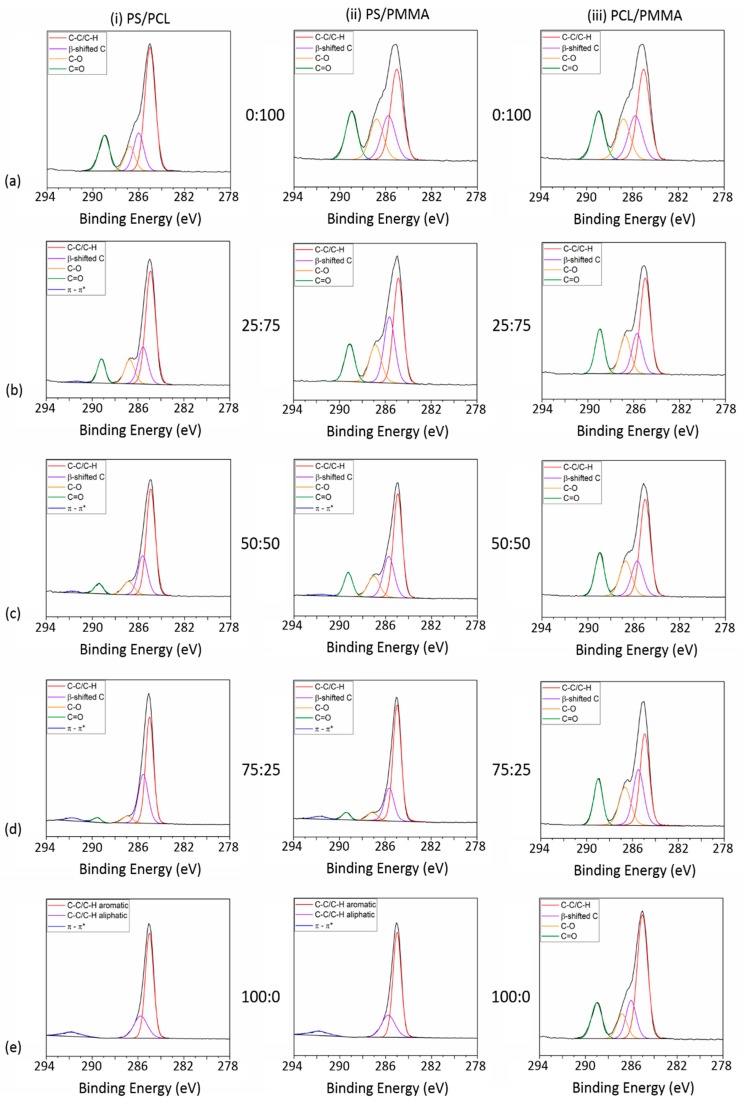
High resolution C 1s spectra of (i) PS/PCL, (ii) PS/PMMA and (iii) PCL/PMMA demixed films in varying ratios of (**a**) 0:100, (**b**) 25:75, (**c**) 50:50, (**d**) 75:25 and (**e**) 100:0.

The elemental surface compositions of the polymer demixed films were determined by XPS analysis. The quantitative data is given in Table 2 and the spectra of the C 1s high resolution scans are shown in Figure 2. Curve fitting of the C 1s spectrum for PS_100_ gave three components: aromatic C–C/C–H at 285.0 eV, aliphatic C–C/C–H at 285.6 eV and the π–π* shakeup at 291.7 eV. For both PCL_100_ and PMMA_100_, the C 1s spectra was curve fitted into four components: C–C/C–H at 285.0 eV, β-shifted C at 285.5 eV, C–O at 286.3 eV and C=O at 289.1 eV. In PS / PCL films, the increase in relative PS concentration was indicated by the increase of at.% contribution of the π–π* shakeup from 0.7% in PS_25_PCL_75_ up to 6.2% in PS_100_. The PCL-specific C–O and C=O component contributions decreased from 8.1% and 15.7% to 4.6% and 1.6%, respectively, going from PCL_100_ to PS_75_PCL_25_. In PS/PMMA films, π–π* shakeup was not observed in PS_25_PMMA_75_ but contributed 1.2% of the PS_50_PMMA_50_ C 1s envelope and 2.0% of PS_75_PMMA_25_. The contribution of the PMMA-specific C–O and C=O components decreased from 18.3% (C–O) and 15.2% (C=O) in PS_25_PMMA_75_ to 9.1% (C–O) and 6.5% (C=O) in PS_75_PMMA_25_. In PCL/PMMA films, there was no significant difference in at.% contributions of β-shifted C, C–O and C=O peaks in PCL_25_PMMA_75_, PCL_50_PMMA_50_ and PCL_75_PMMA_25_.

To analyse which polymer segregates to the air interface, the relative contribution of the ester (C=O) component to the C 1s envelope in the XPS spectra of the demixed films was compared to the 100% polymer control films, as previously reported by Ton-That and D’Sa [21,35]. In two of the systems studied (PS/PCL and PS/PMMA), the ester functional group is unique to one polymer (PCL and PMMA). As such, it is possible to determine the surface composition of each demixed PS/PCL and PS/PMMA films. In PCL/PMMA demixed films, the ester functional group is present in the backbone of each polymer and this method is unsuitable for this blend system. XPS analysis was carried out on polymer demixed films before and after curing at 80 °C to determine whether the vertical phase separation was thermodynamically stable.

For both PS/PCL and PS/PMMA blend systems the arbitrary term ‘A’ can be defined as the fraction of O–C=O peak in the C 1s spectrum:

(1) $$A=\left(\frac{I_{O-C=O}}{I_{C}}\right)$$

In PS/PCL blends the relative contribution of PCL is 6 *X_PCL_* (six carbon atoms per repeat unit) and in PS/PMMA the relative contribution of PMMA is 5 *X_PMMA_* (five carbon atoms per repeat unit). The relative contribution of PS in both blend systems is 8(1 − *X*) (eight carbon atoms per repeat unit), where *X_n_* is the molar surface concentration of the polymer, to give:

(2) $$A=\frac{X_{PCL}}{6X_{PCL}+8\left(1-X_{PCL}\right)}$$

for PS/PCL blends and,

(3) $$A=\frac{X_{PMMA}}{5X_{PMMA}+8\left(1-X_{PMMA}\right)}$$

for PS/PMMA blends.

Rearranging (2) and (3) gives (4) and (5), respectively:

(4) $$X_{PCL}=\frac{8A}{2A+1}$$

(5) $$X_{PMMA}=\frac{8A}{3A+1}$$

**Figure 3:**
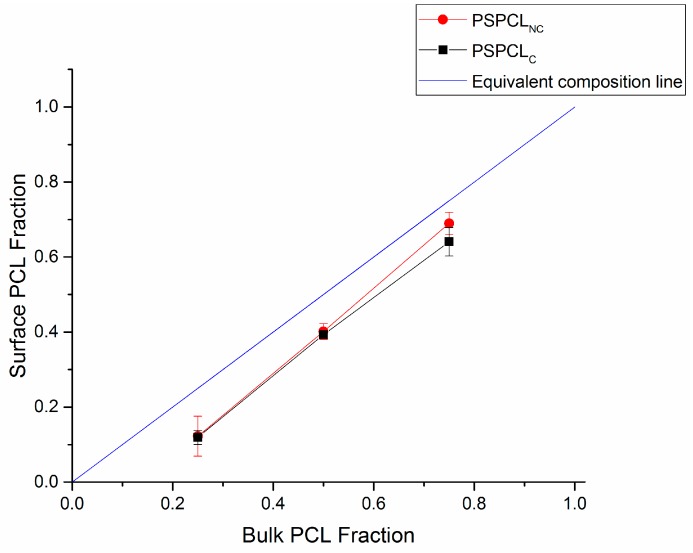
Plot of PS/PCL film surface PCL fraction vs. bulk PCL fraction. Data obtained using high resolution C 1s XPS measurements and Equation (4).

**Figure 4:**
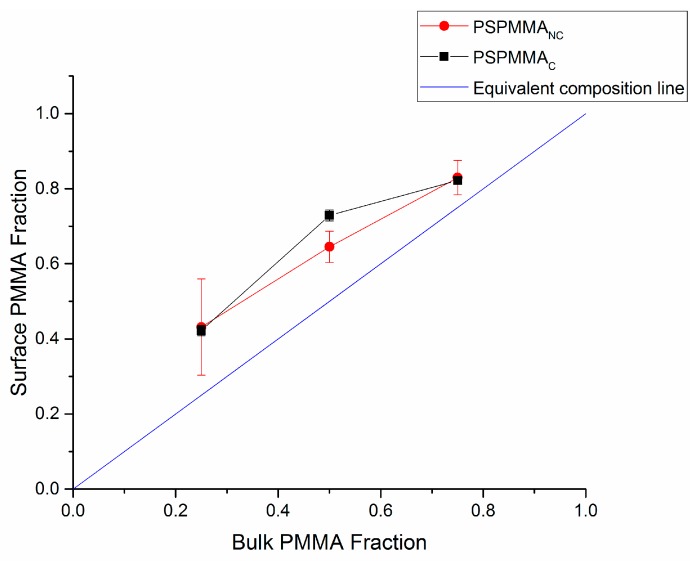
Plot of PS/PMMA film surface PMMA fraction vs. bulk PMMA fraction. Data obtained using high resolution C 1s XPS measurements and Equation (5).

In Figure 3 and Figure 4, the surface fraction of PCL and PMMA in PS/PCL and PS/PMMA demixed films, respectively, deduced from Equations (4) and (5), were compared to the equivalent composition of their corresponding 100% polymer film.

In PS/PCL demixed films, the surface fraction of PCL is below the theoretical equivalent composition line, indicating that PS segregates to the air interface, whereas in PS/PMMA demixed films the PMMA surface fraction is above the theoretical equivalent composition line, indicating that PMMA segregates to the air interface in this system. No movement of data points across the equivalent composition line indicates that the initial kinetic arrangement is also a thermodynamically stable one.

### 3.4. Surface Topography: AFM

**Figure 5:**
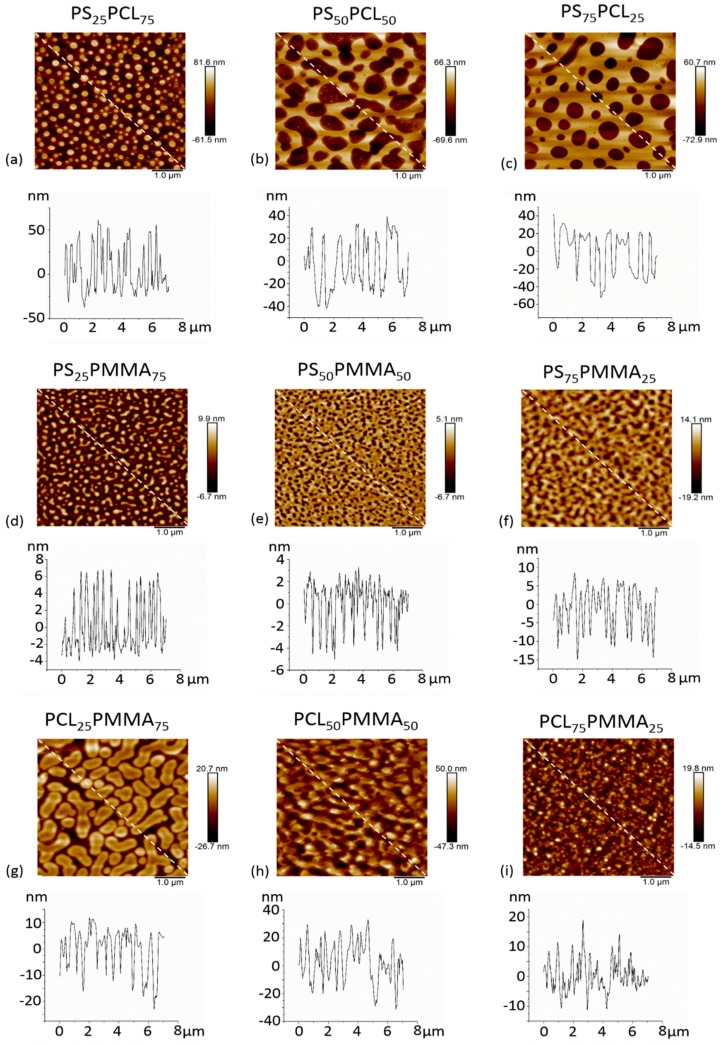
AFM 3D images and depth profiles of (**a**) PS_25_PCL_75_, (**b**) PS_50_PCL_50_, (**c**) PS_75_PCL_25_, (**d**) PS_25_PMMA_75_, (**e**) PS_50_PMMA_50_, (**f**) PS75PMMA_25_, (**g**) PCL_25_PMMA_75_, (**h**) PCL_50_PMMA_50_ and (**i**) PCL_75_PMMA_25_ demixed films.

AFM was used to determine the surface topography of the polymer demixed films and representative height profiles are displayed in Figure 5. AFM derived data including surface features and roughness values (*Rq*, *Ra*) are presented in Table 3. The 100% polymer control films exhibited flat surfaces with no features. The topography of the polymer demixed films was dependent on the blend system and the relative ratios of the polymers. In PS/PCL demixed films, when PCL was in excess an island topography was observed with average feature heights of 48 nm and average feature diameters of 185 nm. PS_50_PCL_50_ demixed films formed ribbon topographies with average peak heights and diameters of 65 nm and 609 nm, respectively. Pits were formed when PS was in abundance and the feature depths averaged 72 nm and the diameters 711 nm. In PS/PMMA demixed films, when PMMA was the more concentrated polymer, islands were formed of average height 7 nm and average diameter 160 nm. For both PS_50_PMMA_50_ and PS_75_PMMA_25_ demixed films a pit topography was displayed with average depths of 8 and 11 nm and diameters of 118 and 190 nm, respectively. In PCL/PMMA demixed films, islands were formed for all polymer ratios. When PMMA was in abundance these islands were depressed in the apex, with average heights of 29 nm and average diameters of 232 nm. When polymers were in equal concentration, islands were formed, 30 nm in height and 154 nm in diameter. The islands decreased in height (by an average of 16 nm) when the PCL concentration increased to 75%, but there was no significant difference between the diameter (on average 162 nm) of the structures.

### 3.5. Bacterial Response: Adhered Cell CFU Assay

**Figure 6:**
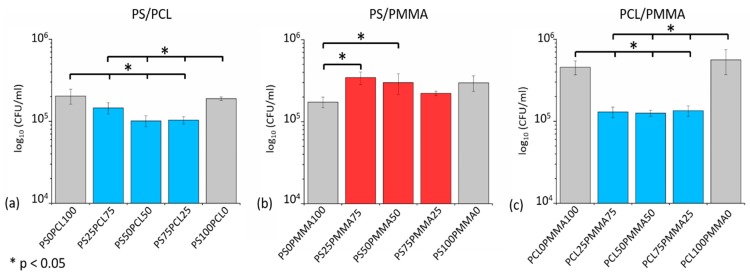
Viable adhered PA14 cell counts (CFU/mL) after 24 h growth on (**a**) PS/PCL, (**b**) PS/PMMA and (**c**) PCL/PMMA. The symbol ‘*’ and blue bars indicate that all polymer demixed films have a statistically significant reduction in cell counts compared to controls (*p < 0.05*). Red bars indicate no statistically significant reduction or a statistically significant increase in cell count. Grey bars are pure polymer control surfaces.

Antibacterial activity was investigated against the lab strain of *P. aeruginosa*, PA14. An adhered cell CFU assay was performed, in which the surfaces were inoculated with bacteria for 24 h to allow any potential biofilm formation. After removal of any planktonic bacteria with a PBS wash, remaining viable bacteria from the surface were counted to test whether materials with structured surface topographies prevented adhesion and therefore biofilm formation. The cell counts for the adhered cell CFU assays are given in Figure 6 for PS/PCL, PS/PMMA and PCL/PMMA demixed films with 100% polymer films as control surfaces. On PS/PCL demixed films (Figure 6a), all bacterial counts were significantly reduced compared to the control surfaces. The PS_25_PCL_75_ film reduced the bacterial count by 28% and 23% with respect to PCL_100_ and PS_100_. For PS_50_PCL_50_, reductions of 50% and 46% were observed for PCL_100_ and PS_100,_ respectively. Finally, on the PS_75_PCL_25_ films the count was 49% less than PCL_100_ and 45% less than PS_100_. In PS/PMMA demixed films (Figure 6b), there was no statistically significant reduction for PA14 adhered cell CFU counts, compared to either PS_100_ or PMMA_100_. All PCL/PMMA demixed films (Figure 6c) exhibited statistically significant reductions in CFU counts compared to PCL_100_ and PMMA_100_. The counts on PCL_25_PMMA_75_ were reduced by 77% compared to on PCL_100_ and 72% compared to on PMMA_100_. For PCL_50_PMMA_50_, reductions of 78% and 73% were observed against PCL_100_ and PMMA_100_, respectively. The counts on PCL_75_PMMA_25_ were reduced by 76% and 70% compared to PCL_100_ and PMMA_100_, respectively.

## 4. Discussion

**Figure 7:**
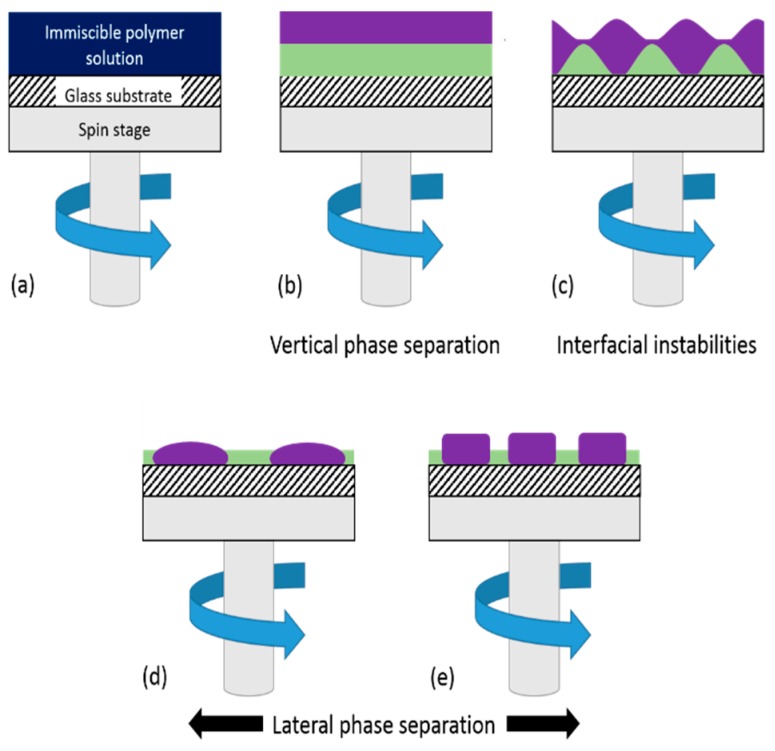
The polymer demixing process: a solution of two immiscible polymers is spun down and after an initial spin-off (**a**) the film splits vertically into two layers (**b**). Interfacial instabilities then occur between the polymer layers (**c**) leading to lateral phase separation (**d**), (**e**) [Adapted from Ref. 36].

Spin coating of demixed blends produces surfaces with discrete topographies and wettabilities. A schematic of the steps involved in polymer demixing is displayed in Figure 7 [36].

The immiscibility of the two constituent polymers within a binary blend system when prepared in a common solvent is key to the phase separation phenomena that occur subsequently. Flory [37,38] and Huggins [39] demonstrated that the entropic law that governs the immiscibility of low molecular weight (LMW) compounds (Δ*G_m_* = Δ*H_m_* − *T*Δ*S_m_*) becomes an almost insignificant contributing factor for high molecular weight (HMW) materials, with an increase in molar mass leading to a decrease in Δ*S_m_* resulting in Δ*G_m_* > 0. Solvent and polymer are removed during the initial spin-off stage leading to formation of a bilayer in which one polymer segregates to the substrate interface and one to the air interface. The initial arrangement is purely kinetic with the least soluble polymer wetting the substrate followed by the more soluble polymer wetting the surface [35,40]. Thermodynamic factors may begin to play a role after time, and the polymer with the lowest surface free energy would be expected to segregate to the air interface to maintain the minimum interfacial energy [41,42], at the same time the higher molecular weight polymer tends to favour the substrate interface so as to avoid potential loss of conformational entropy associated with compression on the film surface [42,43].

In these experiments, the polymers were dissolved in chloroform. The Hildebrand solubility parameters for chloroform, PMMA, PS and PCL are 9.29, 8.80, 8.94 and 10.35, respectively. The closer the values of a polymer to that of its solvent generally indicates better solubility (PS≈PMMA>PCL in chloroform). In PS / PCL blends, the more soluble PS segregates to the surface even after curing at 80 °C, despite having a higher surface energy (PS: 40.7 mN/m, PCL: 30.8 mN/m) [41,44,45] and despite the entropic penalty it incurs being the higher molecular weight component (PS: 280 kDa, PCL: 45 kDa). A possible explanation for this is that the pre-cleaning of the glass substrates renders the surface hydrophilic, thereby having a higher affinity for the more polar PCL [41]. 

While the solubility parameters of PS and PMMA are comparable, it can be assumed that PMMA has better solubility in chloroform from its segregation to the surface of the PS/PMMA films (Figure 4). Ton-That et al. investigated the effect of curing PS/PMMA demixed films after the spin coating process to allow the system to potentially reassemble under thermodynamic control [35]. Indeed, they observed that the initial orientation of the polymers was as a result of kinetics, with PMMA segregating to the air interface. Addition of heat to the system rearranged the polymer films, with PMMA migrating to the substrate interface owing to a higher surface energy.

In PS/PMMA demixed films the XPS analysis shows that PMMA prefers the air interface even after curing, despite having a comparable surface energy to PS (PS: 40.7 mN/m, PMMA: 41.0 mN/m) [44] and higher molecular weight (PS: 280 kDa, PMMA: 380 kDa). It is feasible that the curing conditions may have not been adequate to allow the blend system to reach thermodynamic equilibrium.

In the PCL/PMMA blends the ester component is present in both PCL and PMMA and so the XPS-derived relative ester method was unsuitable to determine surface composition for these blends. It can however, be hypothesised that due to higher solubility of PMMA in chloroform the PCL deposits first on the substrate, which is in agreement with the work carried out by Khattak et al. on PCL/PMMA blends [25]. PMMA has a greater surface energy (PCL: 30.8 mN/m, PMMA: 41.0 mN/m) [45,46] and higher molecular weight (PCL: 45 kDa, PMMA: 380 kDa) so it is hypothesised that the initial kinetic arrangement is most likely thermodynamically unstable and may result in a rearrangement of PCL to the surface.

The spin coating of polymer demixed blends in varying relative concentrations leads to the formation of films with island-, ribbon- and pit-like nanotopographical surface structures. This arises through complex dewetting pathways in which one or both polymer in the blend dewets the underlying surface, leading to lateral phase separation. The dewetting pathway depends on the relative concentrations of the polymers and the nature of the vertical phase separation. The dewetting of the two layers in the system is governed by the dynamics between the deformable polymer-air and polymer-polymer interfaces. An instability is induced between the two interfaces of the less concentrated (thinner) polymer layer [46]. Ma et al. reported on the underlying mechanisms involved in the formation of the surface morphologies seen in PS/PCL demixed films [41]. The report corroborates the findings in this work, observing that vertical separation occurs first with PS segregating to the air interface. They proposed that the thin upper PS layer results in instabilities at the interfaces of the layer, resulting in dewetting of the PS layer from the PCL layer to give PS islands as like for PS_25_PCL_75_. When the PS concentration increased vertical phase separation led to a liquid-liquid dewetting process, in which both films dewet their underlying surface. The PS chains lose mobility as the solvent evaporates and this results in ribbon-like structures as seen on PS_50_PCL_50_ surfaces. When the concentration of PS increased, further dewetting led to the growth of holes, like the pits observed for PS_75_PCL_25_ blends. 

De Silva et al. demonstrated that by varying the concentrations of the polymers in PS/PMMA blends and as a result, the relative thicknesses of the individual polymer layers, the instability can be transferred between layers [47]. It was seen that when the PMMA top layer was thinner than the underlying PS layer the instability was between the interfaces of the PMMA layers. Vice versa, when the PMMA concentration increased (thicker layer) on top of the thinner PS layer a transition to an unstable PS layer was observed. It is, therefore, most likely that the island-rich topography observed on the surface of PS_25_PMMA_75_ films is due to an initial dewetting of PS from the surface, and the pits formed on PS_75_PMMA_25_ most likely due to the growth of holes, initiated by the dewetting of PMMA on the PS layer. The hypothesis that the more soluble PMMA segregates to the air interface corroborates Khattak’s work on PCL/PMMA [25]. From the reports by both Ma and de Silva, it can be concluded that the initial interfacial instability occurs in the lower concentration (thinner) layer [41,47]. In PCL_25_PMMA_75_ the pitted island formation is likely formed through an initial instability in the underlying PCL layer dewetting the substrate, while the island morphology seen for PCL_75_PMMA_25_ begins with an instability in the PMMA layer, dewetting the PCL layer.

In this study, the polymer demixed films were assayed for their anti-adhesion efficacy against *P. aeruginosa* (PA14), using an adhered cell CFU assay to see if biofilm formation could be prevented. An inoculating period of 24 h was chosen to allow the potential for irreversible adhesion and subsequent biofilm formation to occur. A study by Lu et al. in 2016, reported the anti-adhesive properties of polydimethylsiloxane (PDMS) with micropatterned surfaces [48]. A significant reduction in bacterial adhesion was observed for three different species of bacteria (*E. coli*, *S. aureus,* and *P. aeruginosa*). When the pattern size was smaller than the bacterial cell diameter an improved anti-adhesion effect was observed; when the pattern size was comparable or larger than the bacterial cell, the anti-adhesion effect was reduced.

Of the three polymer blend systems used in this study, PS/PCL and PCL/PMMA demixed films showed a significant reduction in bacterial adhesion compared to their corresponding 100% polymer control films. For PS/PCL demixed films PS_50_PCL_50_ and PS_75_PCL_25_ showed increased antibacterial activity compared to PS_25_PCL_75_, whereas there was no statistical significance between cell counts on the surface of the PCL/PMMA demixed films. This is probably due to the fact that topographies exhibited by PCL/PMMA films did not vary in shape and size as drastically as in PS/PCL films. The results corroborate Lu’s [48] findings with the diameters of the nanotopographical surface features of the demixed films being smaller than the experimental diameter of PA14 cells (1.7 ± 0.3 µm) (Supporting information, Figure S2). No reduction in bacterial adhesion was observed for PS/PMMA demixed films when compared to their corresponding 100% polymer control films. After 24 h soaking in LB broth, the diameters of the nanotopographical surface features of all PS/PMMA demixed films (Supporting information, Table S1, Figure S1) had increased to sizes comparable to the bacterial cell diameter. This was also in agreement with Lu’s observations [49] that the anti-adhesion effect is reduced when cell and structure diameters are comparable.

Hochbaum and Aizenberg demonstrated the ability to the affect the spontaneous ordering and arrangement of *P. aeruginosa* cells on nanostructured substrate surfaces by altering the spacing between structures [49]. The bacteria were seen to arrange themselves so as to maximise the cell-surface contact area. When the spacing was larger than the cell diameter, the adhesion of the bacteria on the surface was random; when the spacing between features was comparable to the diameter of the cell, the bacteria positioned themselves parallel to the substrate and perpendicular to one another, in contact with multiple surface structures; when the structures were smaller than the diameter of the cell, the bacteria were no longer able to arrange themselves parallel to the surface and oriented so as to adhere to the vertical length of the structures, perpendicular to the surface. In this study, the spacing between features on the surface of the anti-adhesive PS/PCL and PCL/PMMA demixed films were significantly smaller than the length of the cell. The heights of these surface structures were also shorter than the length of the cell meaning that a vertical arrangement perpendicular to the surface would not benefit the bacteria in maximising cell-surface contact area. As such these two polymer blend systems (PS/PCL, PCL/PMMA) were able to produce topographies that were anti-adhesive to the bacteria by preventing the bacteria from maximising their cell-surface contact area. Future studies within the group are looking using at using different fabrication technologies for generating specific features with well-defined spacings, pitches and heights further understand bacterial adhesion to nanotopographies.

## 5. Conclusions

In summary, we used a cost-effective, polymer demixing protocol to produce binary polymer demixed thin films with varying topographies and wettabilities. The nanotopographical structures varied between islands, ribbons and pits, depending on the polymer blend system used and the relative concentrations of the two polymers, within the blends. The anti-adhesive activity and thus prevention of biofilm formation of the polymer demixed films was assessed against *P. aeruginosa* (PA14) after 24 h. All PS/PCL and PCL/PMMA demixed films showed anti-adhesive properties when compared to their corresponding 100% polymer control films; PS/PMMA demixed films showed no anti-adhesive ability. The link between feature size/spacing distance and cell diameter is in agreement with the conclusions drawn from the literature [48,49]. PS/PCL and PCL/PMMA demixed films were prepared with feature size and spacing between features much smaller than the bacterial cell. As a result, of this the surface was less desirable for the bacteria to adhere to due to the inability to maximise cell-surface contact area.

## Figures and Tables

**Table 1:** Static water contact angle measurements of polymer demixed films (PS/PCL, PS/PMMA, PCL/PMMA) with varying ratios (0:100, 25:75, 50:50, 75:25, 100:0). The results are presented as mean average ± standard deviation. Matching superscript letters in each column indicate that there is a significant difference between the contact angles of these surfaces (*P* < 0.05).

	Contact Angle (^o^)		
	PS:PCL	PS:PMMA	PCL:PMMA
0:100	76.2 ± 1.2 ^a^	68.9 ± 1.6 ^a,f^	68.9 ± 1.6 ^a^
25:75	71.8 ± 3.6 ^b^	70.0 ± 1.0 ^b,c^	67.2 ± 2.4 ^b,c^
50:50	72.9 ± 3.8 ^c,d^	68.6 ± 2.4 ^d,e^	71.4 ± 1.2
75:25	81.4 ± 2.3 ^b,c^	81.1 ± 5.3 ^b,d,f^	72.7 ± 2.8 ^b^
100:0	83.8 ± 1.5 ^a,b,d^	83.8 ± 1.5 ^a,c,e^	76.2 ± 1.2 ^a,c^

**Table 2:** XPS-derived at.% of curve-fitted C 1s components for PS/PCL, PS/PMMA and PCL/PMMA demixed films.

	C 1s Component at.% (Binding Energy, eV)				
	C–C/C–H Aliphatic (285.0)	β-shifted C (C–C/C–H Aromatic) ^a^ (285.5)	C–O (286.3)	C=O (289.1)	π–π* (291.6)
PCL100	56.0 ± 3.9	15.9 ± 0.7	8.1 ± 2.5	15.7 ± 0.6	-
PS25PCL75	54.4 ± 4.4	21.7 ± 4.7	13.1 ± 0.6	10.2 ± 0.3	0.7 ± 0.2
PS50PCL50	69.3 ± 7.5	15.7 ± 7.5	8.4 ± 0.5	5.6 ± 0.5	1.3 ± 0.4
PS75PCL25	55.9 ± 2.9	34.0 ± 2.9	4.6 ± 0.5	1.6 ± 0.7	3.7 ± 0.7
PS100	69.7 ± 0.6	24.1 ± 0.3 ^a^	-	-	6.2 ± 0.3
PMMA100	38.0 ± 1.3	21.0 ± 1.8	21.4 ± 2.0	19.6 ± 1.0	-
PS25PMMA75	43.5 ± 3.1	23.0 ± 4.2	18.3 ± 1.0	15.2 ± 1.0	-
PS50PMMA50	54.7 ± 4.5	18.0 ± 5.7	15.1 ± 1.5	10.8 ± 0.6	1.2 ± 0.1
PS75PMMA25	62.2 ± 3.7	20.2 ± 1.3	9.1 ± 3.5	6.5 ± 2.2	2.0 ± 0.6
PS100	69.7 ± 0.6	24.1 ± 0.3^a^	-	-	6.2 ± 0.3
PMMA100	38.0 ± 1.3	21.0 ± 1.8	21.4 ± 2.0	19.6 ± 1.0	-
PCL25PMMA75	44.5 ± 1.8	18.3 ± 0.6	19.1 ± 0.6	18.4 ± 0.2	-
PCL50PMMA50	44.5 ± 1.8	17.7 ± 0.8	20.0 ± 3.0	17.8 ± 0.4	-
PCL75PMMA25	42.2 ± 4.8	21.4 ± 4.2	19.2 ± 0.7	17.3 ± 0.2	-
PCL100	56.0 ± 3.9	15.9 ± 0.7	8.1 ± 2.5	15.7 ± 0.6	-

^a^ The peak at 285.5 eV for PS100 is due to C-C/C-H aromatic C 1s components.

**Table 3:** Topographical data for PS/PCL, PS/PMMA, PCL/PMMA demixed films determined by AFM.

	Topography	Feature Height/Depth (nm)	Feature Diameter (nm)	Feature Spacing (nm)	*Rq* (nm)	*Ra* (nm)
PCL_100_	flat	-	-	-	5.2 ± 1.4	3.7 ± 0.9
PS_25_PCL_75_	islands	48 ± 20	185 ± 53	197 ± 69	17.3 ± 5.5	14.4 ± 4.5
PS_50_PCL_50_	ribbons	65 ± 14	609 ± 185	381 ± 237	21.3 ± 4.3	18.7 ± 4.1
PS_75_PCL_25_	pits	72 ± 19	711 ± 239	248 ± 152	23.9 ± 4.4	18.9 ± 4.4
PS_100_	flat	-	-	-	0.3 ± 0.0	0.2 ± 0.0
PMMA_100_	flat	-	-	-	0.3 ± 0.1	0.2 ± 0.0
PS_25_PMMA_75_	islands	7 ± 2	160 ± 42	200 ± 69	2.6 ± 0.4	2.2 ± 0.4
PS_50_PMMA_50_	pits	8 ± 5	118 ± 39	60 ± 33	3.2 ± 1.2	2.5 ± 0.9
PS_75_PMMA_25_	pits	11 ± 4	190 ± 57	88 ± 28	3.5 ± 0.9	2.7 ± 0.7
PS_100_	flat	-	-	-	0.3 ± 0.0	0.2 ± 0.0
PMMA_100_	flat	-	-	-	0.3 ± 0.1	0.2 ± 0.0
PCL_25_PMMA_75_	pitted islands	29 ± 12	232 ± 100	145 ± 101	9.8 ± 5.4	8.4 ± 5.0
PCL_50_PMMA_50_	islands	30 ± 20	154 ± 82	191 ± 113	9.6 ± 5.9	7.9 ± 4.7
PCL_75_PMMA_25_	islands	16 ± 5	162 ± 57	186 ± 92	3.9 ± 1.0	2.9 ± 0.8
PCL_100_	flat	-	-	-	5.2 ± 1.4	3.7 ± 0.9

## Supplementary Materials

The following are available online at https://www.mdpi.com/2073-4360/11/12/1921/s1, Table S1. Topographical data for PS/PCL, PS/PMMA, PCL/PMMA demixed films soaked in LB broth for 24 h, determined by AFM. Figure S1. AFM 3D images and depth profiles of (a) PS_25_PCL_75_, (b) PS_50_PCL_50_, (c) PS_75_PCL_25_, (d) PS_25_PMMA_75_, (e) PS_50_PMMA_50_, (f) PS75PMMA_25_, (g) PCL_25_PMMA_75_, (h) PCL_50_PMMA_50_ and (i) PCL_75_PMMA_25_ demixed films, after soaking in LB broth for 24 h. Figure S2. Representative SEM images of PA14 fixed onto glass slides after 24 h incubation time. (Average cell diameter 1.7 ± 0.3 µm).
