# Supplementary material for: Effect of Polymer Demixed Nanotopographies on Bacterial Adhesion and Biofilm Formation
Source: Polymers (Basel). 2019 Nov 21;11(12):1921. doi: 10.3390/polym11121921 (PMC6960884; doi:10.3390/polym11121921)
Supplement: Supplementary file 1 [file polymers-11-01921-s001.pdf]

Effect of Polymer Demixed Nanotopographies on Bacterial Adhesion and Biofilm Formation

George Fleming [1], Jenny Aveyard [1], Joanne L Fothergill [2], Fiona McBride [3] and Rasmita Raval [3] Raechelle A D'Sa [1,*]

[1] School of Engineering, University of Liverpool, Liverpool L69 3GH, UK; sggflemi@liverpool.ac.uk (G.F.); zippy78@liverpool.ac.uk (J.A.)

[2] Institute of Infection and Global Health, University of Liverpool, 8 West Derby Street, Liverpool L69 7B3, UK; jofoth@liverpool.ac.uk

[3] The Open Innovation Hub for Antimicrobial Surfaces, Surface Science Research Centre, University of Liverpool, Liverpool L69 3BX, UK; fmcbride@liverpool.ac.uk (F.M.); raval@liverpool.ac.uk (R.R.)

[*] Correspondence: r.dsa@liverpool.ac.uk

Table S1. Topographical data for PS/PCL, PS/PMMA, PCL/PMMA demixed films soaked in LB broth for 24 hrs, determined by AFM.

	Topography	Feature height/depth (nm)	Feature Diameter (nm)	Feature spacing (nm)	Rq (nm)	Ra (nm)
PCL_{100}	flat	-	-	-	3.4 ± 0.1	2.5 ± 0.1
$PS_{25}PCL_{75}$	islands	52 ± 13	252 ± 91	125 ± 62	5.2 ± 1.4	3.7 ± 0.9
$PS_{50}PCL_{50}$	ribbons	38 ± 8	226 ± 79	197 ± 68	11.7 ± 1.4	9.7 ± 1.2
$PS_{75}PCL_{25}$	pits	32 ± 8	252± 86	220 ± 57	10.4± 4.3	7.9 ± 3.6
PS_{100}	flat	-	-	-	12.9 ± 3.9	7.5 ± 2.3
$PMMA_{100}$	flat	-	-	-	1.7 ± 0.8	1.2 ± 0.4
$PS_{25}PMMA_{75}$	islands	76 ± 12	1530 ± 265	869 ± 512	14.4 ± 11.9	10.3± 8.3
$PS_{50}PMMA_{50}$	pits	16 ± 5	942 ± 403	942 ± 403	4.5 ± 2.1	3.1 ± 2.0
$PS_{75}PMMA_{25}$	islands	91 ± 9	1386 ± 444	891 ± 241	27.6 ± 6.1	22.2 ± 4.1
PS_{100}	flat	-	-	-	12.9 ± 3.9	7.5 ± 2.3
$PMMA_{100}$	flat	-	-	-	1.7 ± 0.8	1.2 ± 0.4
$PCL_{25}PMMA_{75}$	islands	78 ± 28	418 ± 75	163 ± 38	28.8 ± 9.1	22.6 ± 8.2
$PCL_{50}PMMA_{50}$	islands	37 ± 11	176 ± 45	218 ± 105	12.6 ± 0.0	10.2 ± 0.3
$PCL_{75}PMMA_{25}$	islands	88 ± 19	512 ± 224	412 ± 167	27.0 ± 5.3	21.5 ± 4.7
PCL_{100}	flat	-	-	-	3.4 ± 0.1	2.5 ± 0.1

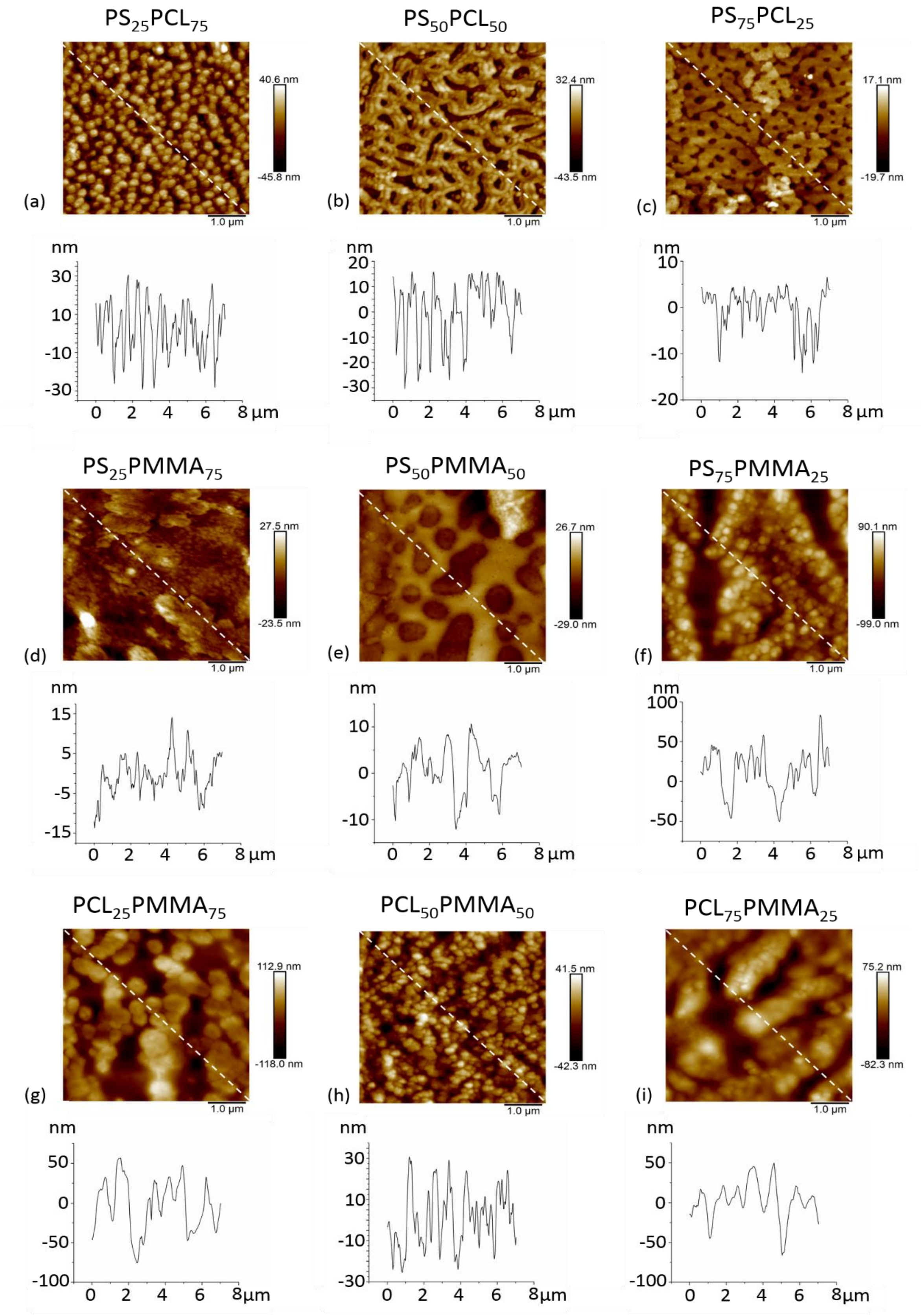

Figure S1. AFM 3D images and depth profiles of (**a**) $PS_{25}PCL_{75}$, (**b**) $PS_{50}PCL_{50}$, (**c**) $PS_{75}PCL_{25}$, (**d**) $PS_{25}PMMA_{75}$, (**e**) $PS_{50}PMMA_{50}$, (**f**) $PS75PMMA_{25}$, (**g**) $PCL_{25}PMMA_{75}$, (**h**) $PCL_{50}PMMA_{50}$ and (**i**) $PCL_{75}PMMA_{25}$ demixed films, after soaking in LB broth for 24 hrs.

SEM

Bacterial cells were fixed to 13 mm glass slides to determine their size. Clean glass coverslips were inoculated for 24 h with 2 ml of bacterial solution (PA14), prepared from an overnight culture and diluted to McFarland Standard 0.5. The bacteria were then fixed with 2.5% glutaraldehyde solution in sterile PBS for 4 hrs. The bacteria were then dehydrated in increasing concentrations of ethanol (30, 50, 75, 90, 95 and 100 v/v %) by soaking for 5 min in each ethanol concentration. The coverslips were dried in air and stored at 4°C before SEM imaging.

Samples were sputter coated with 15 nm coatings of chrome using a Quorum QT150T turbomolecular pumped coater (Quorum Technologies, Lewes, UK). Samples were imaged using a JSM-6610LV SEM system (JEOL USA, Peabody, MA, USA), at working distance of 14 nm and using an accelerating voltage of 10 keV.

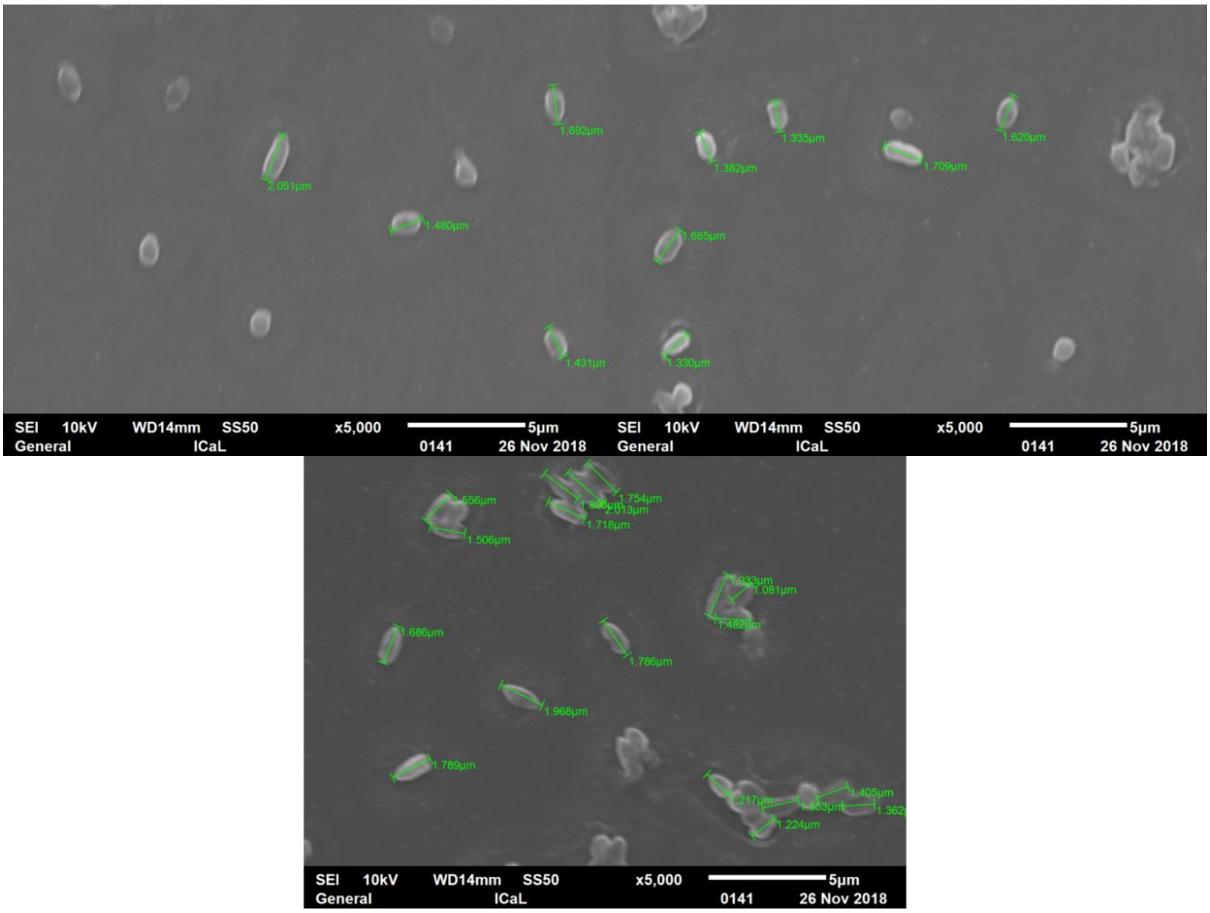

Figure S2. Representative SEM images of PA14 fixed onto glass slides after 24 h incubation time. (Average cell diameter 1.7 ± 0.3 μm).
